# *Resenha sobre o livro *A Pesquisa Científica na Era do Big Data: Cinco Maneiras que Mostram como o Big Data Prejudica a Ciência e como Podemos Salvá-la

**DOI:** 10.1590/0102-311XPT075723

**Published:** 2023-07-17

**Authors:** Saint Clair dos Santos Gomes

**Affiliations:** 1 Instituto Nacional de Saúde da Mulher, da Criança e do Adolescente Fernandes Figueira, Fundação Oswaldo Cruz, Rio de Janeiro, Brasil.

Frases de efeito como “a transformação digital mudou os negócios para sempre” ou “dados se tornaram extremamente valiosos, sendo o novo petróle*o*” são geralmente utilizadas como pano de fundo para a apresentação de soluções baseadas em *big data*^1^. Em geral, essas soluções são desenvolvidas e anunciadas por profissionais da área de tecnologia da informação, reforçando um estereótipo de que os campos das ciências humanas, sociais e da saúde têm pouco, ou nenhum, interesse no assunto.

Nesse sentido, a filósofa Sabina Leonelli, autora do livro *A Pesquisa Científica na Era do Big Data: Cinco Maneiras que Mostram como o Big Data Prejudica a Ciência e como Podemos Salvá-la*^2^, desmistifica essa questão, mostrando que o *big data* é um assunto que impacta todas as áreas do conhecimento humano ^3^^,^^4^. A autora demonstra, a partir de argumentos consistentes, a necessidade cada vez maior de apropriação dos conceitos e aspectos epistemológicos envolvidos no universo da ciência de dados por todos os setores da sociedade ^4^. Ao longo dos seus seis capítulos, percebe-se que a obra foi escrita por uma pessoa apaixonada pelo tema, mas que mantém uma visão crítica e realista das reais possibilidades e limitações de um *big data*. Vários conceitos e termos, por vezes difíceis de serem compreendidos por aqueles não familiarizados com o assunto, são apresentados de forma leve, agradável e compreensiva, porém sempre com o rigor técnico-científico necessário ^5^.

O livro conta com um feliz prefácio de Bethânia Almeida e Mauricio Barreto, que consegue traduzir como a busca para a solução dos desafios enfrentados pelo Centro de Integração de Dados e Conhecimentos para Saúde, Fundação Oswaldo Cruz (Cidacs/Fiocruz Brasília), na integração de grandes volumes de dados, se mostrou uma janela de oportunidades para uma maior aproximação com Sabina Leonelli e toda sua equipe de técnicos e cientistas, surgindo, desse encontro, a inspiração para a elaboração da obra.

O capítulo 1, *O Que É Big Data*, apresenta a conceituação de um *big data* a partir da visão da autora e avança em uma discussão das características esperadas para essa ferramenta, que vão muito além de “muitos dados”. Nesse capítulo também se discute a relação intrínseca, por vezes revolucionária, entre *big data*, dados abertos e a abordagem focada em dados para geração de conhecimento. Os argumentos apresentados levam o leitor a refletir sobre a forma de fazer ciência centrada nos dados ou na teoria e sobre como a concepção inicial de um *big data* pode impactar positivamente e negativamente nos resultados de uma investigação.

No capítulo 2, *Sinais de Alerta: Cinco Maneiras como o Big Data Prejudica a Pesquisa*, a autora apresenta para o leitor os atributos que limitam o uso de um *big data*. São listados itens como: conservadorismo (aqui usado para ilustrar como as decisões e conceituações adotadas para a geração dos dados podem se perpetuar ao longo do tempo, podendo ser completamente inadequadas aos dados mais recentes); insegurança (que, para a autora, se traduz como a possibilidade de avaliação da qualidade e confiabilidade dos dados); mistificação (como um fenômeno decorrente de dados parciais ou pouco representativos); corrupção (como resultado da captação de dados de fontes pouco confiáveis e que alimentam o que se conhece como pós-verdade, geralmente produzidos a partir de fontes que tentam manipular os fatos); e danos sociais (também como fenômenos das fontes pouco confiáveis e que podem gerar resultados que impactam negativamente a sociedade). Esse capítulo é encerrado com uma discussão sobre a ética como parte integrante da ciência, em que é feito um balanço de como os *big data* podem ser potencializadores da ciência e do avanço do conhecimento, assim como podem comprometer, ou mesmo sabotar, a qualidade e a confiabilidade dos achados científicos com impactos importantes na percepção social do valor da ciência.

No capítulo 3, *Como Evitar o Pior: A Abordagem Relacional da Epistemologia do Big Data*, o livro se aprofunda na discussão da necessidade de desenvolvimento de ferramentas que permitam entender a capacidade dos dados de inspirar, corrigir, confirmar ou negar a intuição humana. Com essa motivação, são apresentadas as bases filosóficas para proposições de formas de intervenção na produção, gestão e análise de um *big data*, visando minimizar os riscos decorrentes de conservadorismo, insegurança, mistificação, corrupção e danos sociais descritos no capítulo 2. A autora ainda destaca o papel dos dados nos processos de pesquisa e a construção de conhecimento baseado em dados, defendendo esse tipo de abordagem, porém sem perder a visão crítica consciente.

O livro avança para o capítulo 4, *Como Incentivar o Melhor? Em Direção a uma Ciência Participativa e Responsável*, que retorna a discussão do *big data* como uma ferramenta para combater o fenômeno da pós-verdade e de forças que tentam manipular os fatos. A autora afirma que não há solução mágica ou perfeita para a crise epistêmica dos tempos atuais, decorrente de uma era de tensões e incertezas inerente à multiplicidade de vozes. Segundo Sabina Leonelli, as soluções para melhorar o julgamento dos dados apresentados passam necessariamente por: melhor integração da ética com a pesquisa científica; maior participação social; e desaceleração dos tempos da pesquisa. Nesse capítulo também são exibidos os princípios orientadores para facilitar a transformação do *big data* em conhecimento confiável, tais como: (1) entender o dado como uma categoria relacional; (2) manter uma manutenção regular e de longo prazo da infraestrutura; (3) aperfeiçoar a infraestrutura e habilidade de gerenciamento dos dados; (4) preservar o espaço para a pesquisa exploratória; (5) minimizar os riscos de discriminação e desigualdade com o uso do maior número possível de fontes; (6) ter ética, segurança e responsabilidade social como partes integrantes da pesquisa centrada em dados; (7) vincular o uso do *big data* para fins de pesquisa ao diálogo social; e (8) fomentar o interesse e a disponibilidade de ferramentas para que todos os setores envolvidos em determinado *big data* possam se relacionar e interagir.

Em seu capítulo de conclusão, a autora reforça a tese de que o conhecimento gerado a partir de um *big data* depende das tecnologias envolvidas na produção, no armazenamento e na análise dos dados e que esses atributos são, na verdade, consequência das decisões e escolhas das pessoas responsáveis pela concepção e pelo gerenciamento desses *big data*. Destacam-se como mensagens desse livro que o *big data*, como fonte de geração de conhecimento humano, depende da gestão e confiabilidade dos dados e de como a sociedade interage com esses dados. Além disso, a autora defende veementemente que a pluralidade e a variabilidade dos saberes e métodos do mundo da pesquisa são atributos valiosos que devem ser reconhecidos e explorados como potências que beneficiam o aperfeiçoamento de um *big data*, e não como fonte de complicações por apontarem limitações e problemas.

Em suma, trata-se de uma referência obrigatória para os todos os profissionais, independentemente de suas formações, que desejam ingressar no mundo da ciência de dados, o qual se consolida cada vez mais como uma área do conhecimento humano e já causa grandes impactos e transformações na sociedade.

## References

[1] Quigley E, Holme I, Doyle DM, Ho AK, Ambrose E, Kirkwood K (2021). "Data is the new oil": citizen science and informed consent in an
era of researchers handling of an economically valuable
resource.. Life Sci Soc Policy.

[2] Leonelli S (2022). A pesquisa científica na era do big data: cinco maneiras que mostram
como o big data prejudica a ciência e como podemos salvá-la.

[3] Lasso Cardona LA, Franco Ocampo DF, Estrada Esponda RD (2022). Aplicaciones de la datificación y big data en América Latina
entre el 2015 y 2019. Revista Logos Ciencia & Tecnología.

[4] Saldanha RF, Barcellos C, Pedroso MM (2021). Ciência de dados e big data o que isso significa para estudos
populacionais e da saúde?. Cad Saúde Colet (Rio J.).

[5] Francisco EDR, Kugler JL, Kang SM, Silva R, Whigham PA (2019). Beyond technology management challenges in the big data
era. RAE-Revista de Administração de Empresas.

